# A systematic approach to searching: an efficient and complete method to develop literature searches

**DOI:** 10.5195/jmla.2018.283

**Published:** 2018-10-01

**Authors:** Wichor M. Bramer, Gerdien B. de Jonge, Melissa L. Rethlefsen, Frans Mast, Jos Kleijnen

**Affiliations:** Biomedical Information Specialist, Medical Library, Erasmus MC–Erasmus University Medical Centre, Rotterdam, The Netherlands; Medical Library, Erasmus MC–Erasmus University Medical Centre, Rotterdam, The Netherlands; Spencer S. Eccles Health Sciences Library, University of Utah, Salt Lake City, UT; Medical Library, Erasmus MC–Erasmus University Medical Centre, Rotterdam, The Netherlands; Department of Family Medicine, School for Public Health and Primary Care (CAPHRI), Maastricht University, Maastricht, The Netherlands, and Kleijnen Systematic Reviews, York, United Kingdom

## Abstract

Creating search strategies for systematic reviews, finding the best balance between sensitivity and specificity, and translating search strategies between databases is challenging. Several methods describe standards for systematic search strategies, but a consistent approach for creating an exhaustive search strategy has not yet been fully described in enough detail to be fully replicable. The authors have established a method that describes step by step the process of developing a systematic search strategy as needed in the systematic review. This method describes how single-line search strategies can be prepared in a text document by typing search syntax (such as field codes, parentheses, and Boolean operators) before copying and pasting search terms (keywords and free-text synonyms) that are found in the thesaurus. To help ensure term completeness, we developed a novel optimization technique that is mainly based on comparing the results retrieved by thesaurus terms with those retrieved by the free-text search words to identify potentially relevant candidate search terms. Macros in Microsoft Word have been developed to convert syntaxes between databases and interfaces almost automatically. This method helps information specialists in developing librarian-mediated searches for systematic reviews as well as medical and health care practitioners who are searching for evidence to answer clinical questions. The described method can be used to create complex and comprehensive search strategies for different databases and interfaces, such as those that are needed when searching for relevant references for systematic reviews, and will assist both information specialists and practitioners when they are searching the biomedical literature.

## INTRODUCTION

Librarians and information specialists are often involved in the process of preparing and completing systematic reviews (SRs), where one of their main tasks is to identify relevant references to include in the review [1]. Although several recommendations for the process of searching have been published [2–6], none describe the development of a systematic search strategy from start to finish.

Traditional methods of SR search strategy development and execution are highly time consuming, reportedly requiring up to 100 hours or more [7, 8]. The authors wanted to develop systematic and exhaustive search strategies more efficiently, while preserving the high sensitivity that SR search strategies necessitate. In this article, we describe the method developed at Erasmus University Medical Center (MC) and demonstrate its use through an example search. The efficiency of the search method and outcome of 73 searches that have resulted in published reviews are described in a separate article [9].

As we aimed to describe the creation of systematic searches in full detail, the method starts at a basic level with the analysis of the research question and the creation of search terms. Readers who are new to SR searching are advised to follow all steps described. More experienced searchers can consider the basic steps to be existing knowledge that will already be part of their normal workflow, although step 4 probably differs from general practice. Experienced searchers will gain the most from reading about the novelties in the method as described in steps 10–13 and comparing the examples given in the supplementary appendix to their own practice.

## CREATING A SYSTEMATIC SEARCH STRATEGY

Our methodology for planning and creating a multi-database search strategy consists of the following steps:

1. Determine a clear and focused question
2. Describe the articles that can answer the question
3. Decide which key concepts address the different elements of the question
4. Decide which elements should be used for the best results
5. Choose an appropriate database and interface to start with
6. Document the search process in a text document
7. Identify appropriate index terms in the thesaurus of the first database
8. Identify synonyms in the thesaurus
9. Add variations in search terms
10. Use database-appropriate syntax, with parentheses, Boolean operators, and field codes
11. Optimize the search
12. Evaluate the initial results
13. Check for errors
14. Translate to other databases
15. Test and reiterate

Each step in the process is reflected by an example search described in the supplementary appendix.

### 1. Determine a clear and focused question

A systematic search can best be applied to a well-defined and precise research or clinical question. Questions that are too broad or too vague cannot be answered easily in a systematic way and will generally result in an overwhelming number of search results. On the other hand, a question that is too specific will result into too few or even zero search results. Various papers describe this process in more detail [10–12].

### 2. Describe the articles that can answer the question

Although not all clinical or research questions can be answered in the literature, the next step is to presume that the answer can indeed be found in published studies. A good starting point for a search is hypothesizing what the research that can answer the question would look like. These hypothetical (when possible, combined with known) articles can be used as guidance for constructing the search strategy.

### 3. Decide which key concepts address the different elements of the question

Key concepts are the topics or components that the desired articles should address, such as diseases or conditions, actions, substances, settings, domains (e.g., therapy, diagnosis, etiology), or study types. Key concepts from the research question can be grouped to create elements in the search strategy.

Elements in a search strategy do not necessarily follow the patient, intervention, comparison, outcome (PICO) structure or any other related structure. Using the PICO or another similar framework as guidance can be helpful to consider, especially in the inclusion and exclusion review stage of the SR, but this is not necessary for good search strategy development [13–15]. Sometimes concepts from different parts of the PICO structure can be grouped together into one search element, such as when the desired outcome is frequently described in a certain study type.

### 4. Decide which elements should be used for the best results

Not all elements of a research question should necessarily be used in the search strategy. Some elements are less important than others or may unnecessarily complicate or restrict a search strategy. Adding an element to a search strategy increases the chance of missing relevant references. Therefore, the number of elements in a search strategy should remain as low as possible to optimize recall.

**Figure 1:**
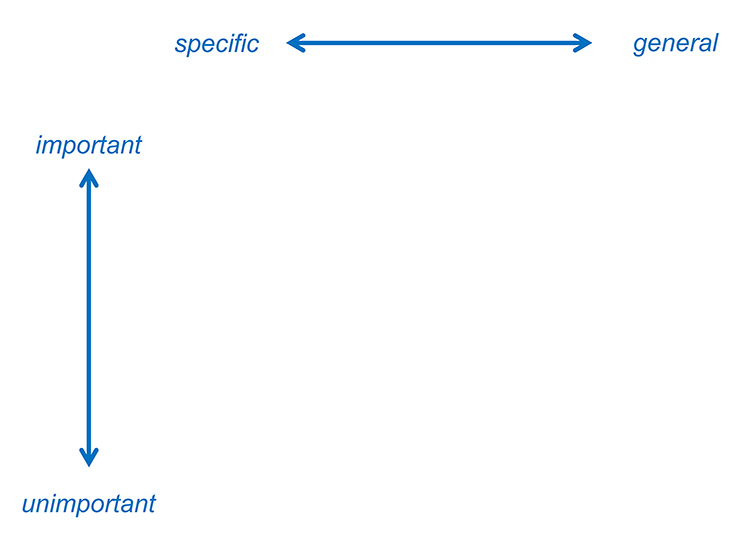
Schema for determining the optimal order of elements

Using the schema in Figure 1, elements can be ordered by their specificity and importance to determine the best search approach. Whether an element is more specific or more general can be measured objectively by the number of hits retrieved in a database when searching for a key term representing that element. Depending on the research question, certain elements are more important than others. If articles (hypothetically or known) exist that can answer the question but lack a certain element in their titles, abstracts, or keywords, that element is unimportant to the question. An element can also be unimportant because of expected bias or an overlap with another element.

#### Bias in elements

The choice of elements in a search strategy can introduce bias through use of overly specific terminology or terms often associated with positive outcomes. For the question “does prolonged breastfeeding improve intelligence outcomes in children?,” searching specifically for the element of *duration* will introduce bias, as articles that find a positive effect of prolonged breastfeeding will be much more likely to mention time factors in their titles or abstracts.

#### Overlapping elements

Elements in a question sometimes overlap in their meaning. Sometimes certain therapies are interventions for one specific disease. The Lichtenstein technique, for example, is a repair method for inguinal hernias. There is no need to include an element of “inguinal hernias” to a search for the effectiveness of the Lichtenstein therapy. Likewise, sometimes certain diseases are only found in certain populations. Adding such an overlapping element could lead to missing relevant references.

The elements to use in a search strategy can be found in the plot of elements in Figure 1, by following the top row from left to right. For this method, we recommend starting with the most important and specific elements. Then, continue with more general and important elements until the number of results is acceptable for screening. Determining how many results are acceptable for screening is often a matter of negotiation with the SR team.

### 5. Choose an appropriate database and interface to start with

Important factors for choosing databases to use are the coverage and the presence of a thesaurus. For medically oriented searches, the coverage and recall of Embase, which includes the MEDLINE database, are superior to those of MEDLINE [16]. Each of these two databases has its own thesaurus with its own unique definitions and structure. Because of the complexity of the Embase thesaurus, Emtree, which contains much more specific thesaurus terms than the MEDLINE Medical Subject Headings (MeSH) thesaurus, translation from Emtree to MeSH is easier than the other way around. Therefore, we recommend starting in Embase.

MEDLINE and Embase are available through many different vendors and interfaces. The choice of an interface and primary database is often determined by the searcher’s accessibility. For our method, an interface that allows searching with proximity operators is desirable, and full functionality of the thesaurus, including explosion of narrower terms, is crucial. We recommend developing a personal workflow that always starts with one specific database and interface.

### 6. Document the search process in a text document

We advise designing and creating the complete search strategies in a log document, instead of directly in the database itself, to register the steps taken and to make searches accountable and reproducible. The developed search strategies can be copied and pasted into the desired databases from the log document. This way, the searcher is in control of the whole process. Any change to the search strategy should be done in the log document, assuring that the search strategy in the log is always the most recent.

### 7. Identify appropriate index terms in the thesaurus of the first database

Searches should start by identifying appropriate thesaurus terms for the desired elements. The thesaurus of the database is searched for matching index terms for each key concept. We advise restricting the initial terms to the most important and most relevant terms. Later in the process, more general terms can be added in the optimization process, in which the effect on the number of hits, and thus the desirability of adding these terms, can be evaluated more easily.

Several factors can complicate the identification of thesaurus terms. Sometimes, one thesaurus term is found that exactly describes a specific element. In contrast, especially in more general elements, multiple thesaurus terms can be found to describe one element. If no relevant thesaurus terms have been found for an element, free-text terms can be used, and possible thesaurus terms found in the resulting references can be added later (step 11).

Sometimes, no distinct thesaurus term is available for a specific key concept that describes the concept in enough detail. In Emtree, one thesaurus term often combines two or more elements. The easiest solution for combining these terms for a sensitive search is to use such a thesaurus term in all elements where it is relevant. Examples are given in the supplementary appendix.

### 8. Identify synonyms in the thesaurus

Most thesauri offer a list of synonyms on their term details page (named *Synonyms* in Emtree and *Entry Terms* in MeSH). To create a sensitive search strategy for SRs, these terms need to be searched as free-text keywords in the title and abstract fields, in addition to searching their associated thesaurus terms.

The Emtree thesaurus contains more synonyms (300,000) than MeSH does (220,000) [17]. The difference in number of terms is even higher considering that many synonyms in MeSH are permuted terms (i.e., inversions of phrases using commas).

Thesaurus terms are ordered in a tree structure. When searching for a more general thesaurus term, the more specific (narrower) terms in the branches below that term will also be searched (this is frequently referred to as “exploding” a thesaurus term). However, to perform a sensitive search, all relevant variations of the narrower terms must be searched as free-text keywords in the title or abstract, in addition to relying on the exploded thesaurus term. Thus, all articles that describe a certain narrower topic in their titles and abstracts will already be retrieved before MeSH terms are added.

### 9. Add variations in search terms (e.g., truncation, spelling differences, abbreviations, opposites)

Truncation allows a searcher to search for words beginning with the same word stem. A search for therap* will, thus, retrieve therapy, therapies, therapeutic, and all other words starting with “therap.” Do not truncate a word stem that is too short. Also, limitations of interfaces should be taken into account, especially in PubMed, where the number of search term variations that can be found by truncation is limited to 600.

Databases contain references to articles using both standard British and American English spellings. Both need to be searched as free-text terms in the title and abstract. Alternatively, many interfaces offer a certain code to replace zero or one characters, allowing a search for “pediatric” or “paediatric” as “p?ediatric.” Table 1 provides a detailed description of the syntax for different interfaces.

**Table 1:** Field codes in five most used interfaces for biomedical literature searching

	PubMed	Ovid	EBSCOhost	Embase.com	ProQuest
Title/abstract	[tiab]^1^	().ab,ti.	TI () OR AB ()^2^	():ab,ti	AB,TI()
All fields	[All Fields]	.af.	2	3	ALL
Thesaurus term	[mesh:noexp]	…/	MH “…”	‘…’/de	MESH(…)
Including narrower	[mesh]	exp …/	MH “…+”	‘…’/exp	MESH#(…)
Combined subheading	*…/sh*[mesh]	exp …/*sh*	MH “…+/*sh*”	‘…’/exp/dm_*sh*^4^	MESH(… LNK ..)
Free subheading	[sh]^5^	.xs. or .fs.^5^	MW	:lnk^5^	
Publication type	[pt]^6^	.pt. or exp *…*/7	PT	:it^6^	RTYPE
Proximity	6	ADJn	Nn	NEAR/n-NEXT/n	N/n
Exact phrase	“double quotes”	No quotes needed	“double quotes”	‘single quotes’	“double quotes”
Truncated phrase	Use-hyphen*	No quote*	No quote*	‘single quote*’	“Double quote*”
Truncation	End	End/ mid	End/ mid	End/ mid	End / mid / start
Infinite	*	* or $	*	*	*
0 or 1 character	—	?	#	—	$1
1 character	—	#	?	?^8^	?
Added to database since	yyyy/mm/dd:yyyy/mm/dd [edat]^9^ (or [mhda])	limit #N to rd=yyyymmdd-yyyymmdd^10^	EM yyyymmdd-yyyymmdd	[dd-mm-yyyy]/sd	LUPD(yyyymmdd)
Publication period (years)	yyyy:yyyy[dp]	limit #N to yr=yyyy-yyyy^11^	PY yyyy-yyyy	[yyyy-yyyy]/py	YR (yyyy-yyyy)
Record sets	#1	1^11^	S1	#1	S1

1 In PubMed, [tiab] should be placed after each search term.

2 EBSCOhost does not allow a combination of fields; all search terms for the title field need to be repeated for the abstract field.

3 EBSCOhost and Embase.com do not use an “all fields” code; a term without a field code is searched in all fields.

4 Subheadings in Embase.com are only applied to diseases (/dm_), drugs (/dd_), or devices (/dv_).

5 [sh] and .xs. include narrower terms for subheadings; .fs. and :lnk do not.

6 In PubMed, proximity searching is not available; search the exact phrase (truncated or between double quotes) or use the Boolean “AND” combination.

7 [pt] and exp …/ includes narrower publication types; .pt. and :it do not.

8 The question mark does not work in combination with field codes.

9 The field [edat] refers to the entry date, when the record was added to PubMed. [mhda] refers to the MeSH date, when the record was last edited.

10 Adding a date limit can only be applied in a separate record set.

11 If a number is to be searched in the text, it should be put between double quotes (e.g., “1”).

Searching for abbreviations can identify extra, relevant references and retrieve more irrelevant ones. The search can be more focused by combining the abbreviation with an important word that is relevant to its meaning or by using the Boolean “NOT” to exclude frequently observed, clearly irrelevant results. We advise that searchers do not exclude all possible irrelevant meanings, as it is very time consuming to identify all the variations, it will result in unnecessarily complicated search strategies, and it may lead to erroneously narrowing the search and, thereby, reduce recall.

Searching partial abbreviations can be useful for retrieving relevant references. For example, it is very likely that an article would mention *osteoarthritis (OA)* early in the abstract, replacing all further occurrences of *osteoarthritis* with *OA*. Therefore, it may not contain the phrase “hip osteoarthritis” but only “hip oa.”

It is also important to search for the opposites of search terms to avoid bias. When searching for “disease recurrence,” articles about “disease free” may be relevant as well. When the desired outcome is *survival*, articles about *mortality* may be relevant.

### 10. Use database-appropriate syntax, with parentheses, Boolean operators, and field codes

Different interfaces require different syntaxes, the special set of rules and symbols unique to each database that define how a correctly constructed search operates. Common syntax components include the use of parentheses and Boolean operators such as “AND,” “OR,” and “NOT,” which are available in all major interfaces. An overview of different syntaxes for four major interfaces for bibliographic medical databases (PubMed, Ovid, EBSCOhost, Embase.com, and ProQuest) is shown in Table 1.

Creating the appropriate syntax for each database, in combination with the selected terms as described in steps 7–9, can be challenging. Following the method outlined below simplifies the process:

- Create single-line queries in a text document (not combining multiple record sets), which allows immediate checking of the relevance of retrieved references and efficient optimization.
- Type the syntax (Boolean operators, parentheses, and field codes) before adding terms, which reduces the chance that errors are made in the syntax, especially in the number of parentheses.
- Use predefined proximity structures including parentheses, such as (() ADJ3 ()) in Ovid, that can be reused in the query when necessary.
- Use thesaurus terms separately from free-text terms of each element. Start an element with all thesaurus terms (using “OR”) and follow with the free-text terms. This allows the unique optimization methods as described in step 11.
- When adding terms to an existing search strategy, pay close attention to the position of the cursor. Make sure to place it appropriately either in the thesaurus terms section, in the title/abstract section, or as an addition (broadening) to an existing proximity search.

The supplementary appendix explains the method of building a query in more detail, step by step for different interfaces: PubMed, Ovid, EBSCOhost, Embase.com, and ProQuest. This method results in a basic search strategy designed to retrieve some relevant references upon which a more thorough search strategy can be built with optimization such as described in step 11.

### 11. Optimize the search

The most important question when performing a systematic search is whether all (or most) potentially relevant articles have been retrieved by the search strategy. This is also the most difficult question to answer, since it is unknown which and how many articles are relevant. It is, therefore, wise first to broaden the initial search strategy, making the search more sensitive, and then check if new relevant articles are found by comparing the set results (i.e., search for Strategy #2 NOT Strategy #1 to see the unique results).

A search strategy should be tested for completeness. Therefore, it is necessary to identify extra, possibly relevant search terms and add them to the test search in an OR relationship with the already used search terms. A good place to start, and a well-known strategy, is scanning the top retrieved articles when sorted by relevance, looking for additional relevant synonyms that could be added to the search strategy.

We have developed a unique optimization method that has not been described before in the literature. This method often adds valuable extra terms to our search strategy and, therefore, extra, relevant references to our search results. Extra synonyms can be found in articles that have been assigned a certain set of thesaurus terms but that lack synonyms in the title and/or abstract that are already present in the current search strategy. Searching for *thesaurus terms NOT free-text terms* will help identify missed free-text terms in the title or abstract. Searching for *free-text terms NOT thesaurus terms* will help identify missed thesaurus terms. If this is done repeatedly for each element, leaving the rest of the query unchanged, this method will help add numerous relevant terms to the query. These steps are explained in detail for five different search platforms in the supplementary appendix.

### 12. Evaluate the initial results

The results should now contain relevant references. If the interface allows relevance ranking, use that in the evaluation. If you know some relevant references that should be included in the research, search for those references specifically; for example, combine a specific (first) author name with a page number and the publication year. Check whether those references are retrieved by the search. If the known relevant references are not retrieved by the search, adapt the search so that they are. If it is unclear which element should be adapted to retrieve a certain article, combine that article with each element separately.

Different outcomes are desired for different types of research questions. For instance, in the case of clinical question answering, the researcher will not be satisfied with many references that contain a lot of irrelevant references. A clinical search should be rather specific and is allowed to miss a relevant reference. In the case of an SR, the researchers do not want to miss any relevant reference and are willing to handle many irrelevant references to do so. The search for references to include in an SR should be very sensitive: no included reference should be missed. A search that is too specific or too sensitive for the intended goal can be adapted to become more sensitive or specific. Steps to increase sensitivity or specificity of a search strategy can be found in the supplementary appendix.

### 13. Check for errors

Errors might not be easily detected. Sometimes clues can be found in the number of results, either when the number of results is much higher or lower than expected or when many retrieved references are not relevant. However, the number expected is often unknown, and very sensitive search strategies will always retrieve many irrelevant articles. Each query should, therefore, be checked for errors.

One of the most frequently occurring errors is missing the Boolean operator “OR.” When no “OR” is added between two search terms, many interfaces automatically add an “AND,” which unintentionally reduces the number of results and likely misses relevant references. One good strategy to identify missing “OR”s is to go to the web page containing the full search strategy, as translated by the database, and using Ctrl-F search for “AND.” Check whether the occurrences of the “AND” operator are deliberate.

Ideally, search strategies should be checked by other information specialists [18]. The Peer Review of Electronic Search Strategies (PRESS) checklist offers good guidance for this process [4]. Apart from the syntax (especially Boolean operators and field codes) of the search strategy, it is wise to have the search terms checked by the clinician or researcher familiar with the topic. At Erasmus MC, researchers and clinicians are involved during the complete process of structuring and optimizing the search strategy. Each word is added after the combined decision of the searcher and the researcher, with the possibility of directly comparing results with and without the new term.

### 14. Translate to other databases

To retrieve as many relevant references as possible, one has to search multiple databases. Translation of complex and exhaustive queries between different databases can be very time consuming and cumbersome. The single-line search strategy approach detailed above allows quick translations using the find and replace method in Microsoft Word (<Ctrl-H>).

**Figure 2:**
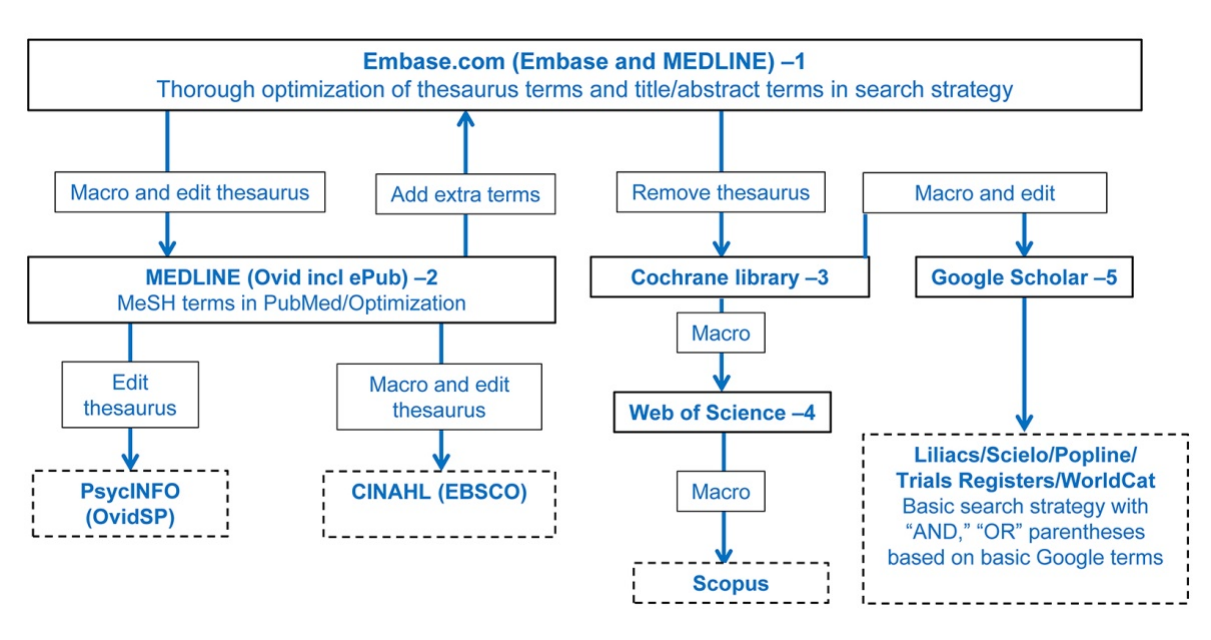
Schematic representation of translation between databases used at Erasmus University Medical Center Dotted lines represent databases that are used in less than 80% of the searches.

At Erasmus MC, macros based on the find-and-replace method in Microsoft Word have been developed for easy and fast translation between the most used databases for biomedical and health sciences questions. The schema that is followed for the translation between databases is shown in Figure 2. Most databases simply follow the structure set by the Embase.com search strategy. The translation from Emtree terms to MeSH terms for MEDLINE in Ovid often identifies new terms that need to be added to the Embase.com search strategy before the translation to other databases.

Using five different macros, a thoroughly optimized query in Embase.com can be relatively quickly translated into eight major databases. Basic search strategies will be created to use in many, mostly smaller, databases, because such niche databases often do not have extensive thesauri or advanced syntax options. Also, there is not much need to use extensive syntax because the number of hits and, therefore, the amount of noise in these databases is generally low. In MEDLINE (Ovid), PsycINFO (Ovid), and CINAHL (EBSCOhost), the thesaurus terms must be adapted manually, as each database has its own custom thesaurus. These macros and instructions for their installation, use, and adaptation are available at bit.ly/databasemacros.

### 15. Test and reiterate

Ideally, exhaustive search strategies should retrieve all references that are covered in a specific database. For SR search strategies, checking searches for their recall is advised. This can be done after included references have been determined by the authors of the systematic review. If additional papers have been identified through other non-database methods (i.e., checking references in included studies), results that were not identified by the database searches should be examined. If these results were available in the databases but not located by the search strategy, the search strategy should be adapted to try to retrieve these results, as they may contain terms that were omitted in the original search strategies. This may enable the identification of additional relevant results.

## DISCUSSION

A methodology for creating exhaustive search strategies has been created that describes all steps of the search process, starting with a question and resulting in thorough search strategies in multiple databases. Many of the steps described are not new, but together, they form a strong method creating high-quality, robust searches in a relatively short time frame.

Our methodology is intended to create thoroughness for literature searches. The optimization method, as described in step 11, will identify missed synonyms or thesaurus terms, unlike any other method that largely depends on predetermined keywords and synonyms. Using this method results in a much quicker search process, compared to traditional methods, especially because of the easier translation between databases and interfaces (step 13). The method is not a guarantee for speed, since speed depends on many factors, including experience. However, by following the steps and using the tools as described above, searchers can gain confidence first and increase speed through practice.

### What is new?

This method encourages searchers to start their search development process using empty syntax first and later adding the thesaurus terms and free-text synonyms. We feel this helps the searcher to focus on the search terms, instead of on the structure of the search query. The optimization method in which new terms are found in the already retrieved articles is used in some other institutes as well but has to our knowledge not been described in the literature. The macros to translate search strategies between interfaces are unique in this method.

### What is different compared to common practice?

Traditionally, librarians and information specialists have focused on creating complex, multi-line (also called line-by-line) search strategies, consisting of multiple record sets, and this method is frequently advised in the literature and handbooks [2, 19–21]. Our method, instead, uses single-line searches, which is critical to its success. Single-line search strategies can be easily adapted by adding or dropping a term without having to recode numbers of record sets, which would be necessary in multi-line searches. They can easily be saved in a text document and repeated by copying and pasting for search updates. Single-line search strategies also allow easy translation to other syntaxes using find-and-replace technology to update field codes and other syntax elements or using macros (step 13).

When constructing a search strategy, the searcher might experience that certain parentheses in the syntax are unnecessary, such as parentheses around all search terms in the title/abstract portion, if there is only one such term, there are double parentheses in the proximity statement, or one of the word groups exists for only one word. One might be tempted to omit those parentheses for ease of reading and management. However, during the optimization process, the searcher is likely to find extra synonyms that might consist of one word. To add those terms to the first query (with reduced parentheses) requires adding extra parentheses (meticulously placing and counting them), whereas, in the latter search, it only requires proper placement of those terms.

Many search methods highly depend on the PICO framework. Research states that often PICO or PICOS is not suitable for every question [22, 23]. There are other acronyms than PICO—such as sample, phenomenon of interest, design, evaluation, research type (SPIDER) [24]—but each is just a variant. In our method, the most important and specific elements of a question are being analyzed for building the best search strategy.

Though it is generally recommended that searchers search both MEDLINE and Embase, most use MEDLINE as the starting point. It is considered the gold standard for biomedical searching, partially due to historical reasons, since it was the first of its kind, and more so now that it is freely available via the PubMed interface. Our method can be used with any database as a starting point, but we use Embase instead of MEDLINE or another database for a number of reasons. First, Embase provides both unique content and the complete content of MEDLINE. Therefore, searching Embase will be, by definition, more complete than searching MEDLINE only. Second, the number of terms in Emtree (the Embase thesaurus) is three times as high as that of MeSH (the MEDLINE thesaurus). It is easier to find MeSH terms after all relevant Emtree terms have been identified than to start with MeSH and translate to Emtree.

At Erasmus MC, the researchers sit next to the information specialist during most of the search strategy design process. This way, the researchers can deliver immediate feedback on the relevance of proposed search terms and retrieved references. The search team then combines knowledge about databases with knowledge about the research topic, which is an important condition to create the highest quality searches.

### Limitations of the method

One disadvantage of single-line searches compared to multi-line search strategies is that errors are harder to recognize. However, with the methods for optimization as described (step 11), errors are recognized easily because missed synonyms and spelling errors will be identified during the process. Also problematic is that more parentheses are needed, making it more difficult for the searcher and others to assess the logic of the search strategy. However, as parentheses and field codes are typed before the search terms are added (step 10), errors in parentheses can be prevented.

Our methodology works best if used in an interface that allows proximity searching. It is recommended that searchers with access to an interface with proximity searching capabilities select one of those as the initial database to develop and optimize the search strategy. Because the PubMed interface does not allow proximity searches, phrases or Boolean “AND” combinations are required. Phrase searching complicates the process and is more specific, with the higher risk of missing relevant articles, and using Boolean “AND” combinations increases sensitivity but at an often high loss of specificity. Due to some searchers’ lack of access to expensive databases or interfaces, the freely available PubMed interface may be necessary to use, though it should never be the sole database used for an SR [2, 16, 25]. A limitation of our method is that it works best with subscription-based and licensed resources.

Another limitation is the customization of the macros to a specific institution’s resources. The macros for the translation between different database interfaces only work between the interfaces as described. To mitigate this, we recommend using the find-and-replace functionality of text editors like Microsoft Word to ease the translation of syntaxes between other databases. Depending on one’s institutional resources, custom macros can be developed using similar methods.

### Results of the method

Whether this method results in exhaustive searches where no important article is missed is difficult to determine, because the number of relevant articles is unknown for any topic. A comparison of several parameters of 73 published reviews that were based on a search developed with this method to 258 reviews that acknowledged information specialists from other Dutch academic hospitals shows that the performance of the searches following our method is comparable to those performed in other institutes but that the time needed to develop the search strategies was much shorter than the time reported for the other reviews [9].

## CONCLUSIONS

With the described method, searchers can gain confidence in their search strategies by finding many relevant words and creating exhaustive search strategies quickly. The approach can be used when performing SR searches or for other purposes such as answering clinical questions, with different expectations of the search’s precision and recall. This method, with practice, provides a stepwise approach that facilitates the search strategy development process from question clarification to final iteration and beyond.

## SUPPLEMENTAL FILE

Appendix Steps in the process of systematic literature searching with an example search
