# Supplementary material for: A systematic approach to searching: an efficient and complete method to develop literature searches
Source: J Med Libr Assoc. 2018 Oct 1;106(4):531–41. doi: 10.5195/jmla.2018.283 (PMC6148622; doi:10.5195/jmla.2018.283)
Supplement: Appendix [file jmla-106-531-s001.pdf]

JMLA Journal of the Medical Library Association

SC

Supplemental content to
J Med Libr Assoc. Oct;106(4):dx.doi.org/10.5195/jmla.2018.283
www.jmla.mlanet.org
© Bramer, de Jonge, Rethlefsen, Mast, Kleijnen 2018

A systematic approach to searching: an efficient and complete method to develop literature searches

Wichor M. Bramer; Gerdien B. de Jonge; Melissa L. Rethlefsen, AHIP; Frans Mast; Jos Kleijnen

APPENDIX

Steps in the process of systematic literature searching with an example search

In this supplemental appendix, the authors describe each step in the process of systematic literature searching and provide an example search.

1. Determine a clear and focused question

The question used as an example throughout this article is:

"Does exercise therapy improve the quality of life of patients with hip osteoarthritis compared to total hip replacement?"

2. Describe the articles that can answer the question

Our example question can be answered by clinical articles, such as, but not limited to, prospective, randomized studies that follow up on patients measuring the outcome of exercise therapy for hip osteoarthritis.

3. Decide which key concepts address the different elements of the question

In the example research question, three elements can be identified: one element is exercise therapy. Because we assume we can find our outcomes of interest in clinical studies, but also want to find studies mentioning our outcomes, the key concepts of treatment effectiveness, clinical studies, and quality of life together form one element. The last element is hip osteoarthritis.

4. Decide which elements should be used for the best results

The plot for our research question is shown in Figure 1. The optimal search strategy will contain two elements: hip osteoarthritis and exercise therapy.

Figure 1 Schema for hip osteoarthritis and exercise therapy

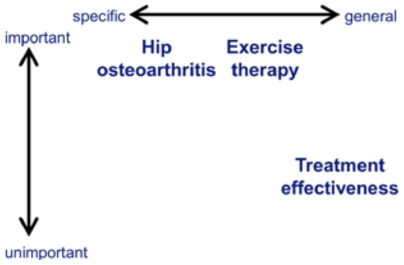

5. Choose an appropriate database and interface to start with

6. Document the search process in a text document

7. Identify appropriate index terms in the thesaurus of the first database

An example of a term for which no thesaurus terms can be found is Bennett's fracture, a fracture of the base of the metacarpal bone of the thumb. It can, in addition to a free-text search for Bennett's fractures, be searched with a combination of the Medical Subject Headings (MeSH) terms ("Fractures, Bone" AND "Metacarpal Bones" AND "Thumb").

An example of a term that combines two concepts is the term "cancer prevention," which is a thesaurus term in Embase. However, "neoplasm" and "prevention" also exist, where prevention is

both an Emtree term and a subheading. The search would then become ('neoplasms'/exp OR 'cancer prevention'/exp) AND ('prevention'/exp OR 'cancer prevention'/exp). Because cancer prevention is a narrower term of prevention, the search can be shortened: ('neoplasms'/exp OR 'cancer prevention'/exp) AND ('prevention'/exp). The syntax used in these examples is that of Embase.com.

Hip Osteoarthritis
MeSH terms: Osteoarthritis, Hip
Emtree terms: hip osteoarthritis, Hip Disability, and Osteoarthritis Outcome Score

Exercise therapy
MeSH terms: Exercise Therapy
Emrtee terms: kinesiotherapy

8. Identify synonyms in the thesaurus

An example of a term with many synonyms is the MeSH term "Observer Variation," which contains among others the entry terms "Observer Bias" and "Interobserver Variability" as well as their inverted counterparts and phrases with additional spaces of hyphens, totaling to up to forty-one different terms and phrases. By using phrase truncation or proximity operators wisely (step 9), the number of search terms needed can be limited; in the case of observer variation, four phrases are sufficient to cover all forty-one entry terms.

An example of a term where narrower terms should be added is Colorectal Neoplasms. Searching with the exploded MeSH term "Colorectal Neoplasms" also retrieves references indexed with the MeSH terms "Colonic Neoplasms," "Rectal Neoplasms," or "Anus Neoplasms." However, in order not to miss any relevant reference, these phrases and their variations should also be searched in title and or abstract.

In the example question, the MeSH term "Osteoarthritis, Hip" displays the Entry Terms "Hip Osteoarthritides"; "Hip Osteoarthritis"; "Osteoarthritides, Hip"; "Coxarthrosis"; "Coxarthroses"; "Osteoarthritis Of Hip"; and "Osteoarthritis Of Hips." To minimize the number of search terms needed and to capitalize on the similarity of the entry terms, we can ignore the inverted term "Osteoarthritides, Hip" and use truncation to search word variants. The final list of collapsed terms in a search interface that does not allow proximity search, such as PubMed, would be Hip Osteoarthrit*, Coxarthros*, and Osteoarthritis of the Hip*.

In addition, before 1989, the disease was indexed with the MeSH terms "Hip, Hip Joint and Osteoarthritis." This means is that older articles can be found by combining either Hip or Hip Joint with Osteoarthritis.

In the branch under the Emtree term kinesiotherapy (the thesaurus term most closely matching the exercise therapy element), many possibly relevant narrower terms are displayed, many of which can be used as free-text search terms (e.g., isokinetic exercise, isometric exercise, movement therapy, and muscle training).

9. Add variations in search terms (e.g., truncation, spelling differences, abbreviations, opposites)

In our example, several variations exist for hip arthritis: hip artheritis, hip arthrosis, etc. However, a search for hip arth* will also find hip arthroplasty, which is not a synonym for hip arthritis. Therefore, we do not recommend that search term.

To reduce the noise observed when searching for an abbreviation such as THA (Total Hop Arthroplasty), several methods can be followed. The term can be combined with a relevant term from its meaning: (THA AND hip) or the searcher could "NOT" out any irrelevant meaning that occurs in the search results: (THA NOT ("Tetracosahexaenoic acid" OR "Threo-hydroxyaspartate" OR "Tetrahydroamentoflavone")).

JMLA Journal of the Medical Library Association

SC
Supplemental content to
J Med Libr Assoc. Oct;106(4):dx.doi.org/10.5195/jmla.2018.283
www.jmla.mlanet.org
© Bramer, de Jonge, Rethlefsen, Mast, Kleijnen 2018

10. Use database-appropriate syntax, with parentheses, Boolean operators, and field codes

Table 1 Using database-appropriate syntax, with parentheses, Boolean operators, and field codes

<table>
<tr><th></th><th>Ovid</th><th>EBSCOhost</th><th>Embase.com</th><th>PubMed sensitive</th><th>PubMed specific</th><th>ProQuest</th></tr>
<tr><td>Prepare a proximity syntax outside of the code to re-use when necessary</td><td>(() ADJ3 ())</td><td>(() N3 ())</td><td>(() NEAR/3 ())</td><td>(() AND ())[1]</td><td></td><td>(() N/3 ())</td></tr>
<tr><td>Start by typing parentheses for the first element</td><td>(|)</td><td>(|)</td><td>(|)</td><td>(|)</td><td>(|)</td><td>(|)</td></tr>
<tr><td>Add standard syntax for thesaurus terms</td><td>(exp "|"/)</td><td>(MH "|+")</td><td>('|'/exp)</td><td>("|" [mh])</td><td>("|"[mh])</td><td>(Mesh# "|")</td></tr>
<tr><td>Paste the relevant thesaurus term(s) in the code</td><td>(exp "Osteoarthritis, Hip"/|)</td><td>(MH "Osteoarthritis, Hip+"|)</td><td>('hip osteoarthritis'/exp|)</td><td>("Osteoarthritis, Hip"[mh] |)</td><td>("Osteoarthritis, Hip"[mh] |)</td><td>(Mesh# "Osteoarthritis, Hip" |)</td></tr>
<tr><td>Add syntax for free-text terms in title abstract</td><td>(exp "Osteoarthritis, Hip"/ OR (|).ab,ti.)</td><td>(MH "Osteoarthritis, Hip+"OR AB (|))[2]</td><td>('hip osteoarthritis'/exp OR (|):ab,ti)</td><td>("Osteoarthritis, Hip"[mh] OR (|))[3]</td><td>("Osteoarthritis, Hip"[mh] OR (|))[3]</td><td>(Mesh# "Osteoarthritis, Hip" OR AB,TI(|))</td></tr>
<tr><td>First, add one word synonyms and exact phrases</td><td>(exp "Osteoarthritis, Hip"/ OR (Coxarthros*|).ab,ti.)</td><td>(MH "Osteoarthritis, Hip+" OR AB (Coxarthros*|))</td><td>('hip osteoarthritis'/exp OR (Coxarthros* OR 'malum coxae sinilis'|):ab,ti)</td><td>("Osteoarthritis, Hip"[mh] OR (Coxarthros*[tiab] |))</td><td>("Osteoarthritis, Hip"[mh] OR (Coxarthros*[tiab] |))</td><td>(Mesh# "Osteoarthritis, Hip" OR AB,TI(Coxarthros*|))</td></tr>
<tr><td>Copy the proximity syntax as created above into the title abstract section</td><td>(exp "Osteoarthritis, Hip"/ OR (Coxarthros* OR ((|) ADJ3 ())).ab,ti.)</td><td>(MH "Osteoarthritis, Hip+" OR AB (Coxarthros* OR ((|) N3 ())))</td><td>('hip osteoarthritis'/exp OR (Coxarthros* OR 'malum coxae sinilis' OR ((|) NEAR/3 ())):ab,ti)</td><td>("Osteoarthritis, Hip"[mh] OR (Coxarthros*[tiab] OR ((|) AND ())))</td><td>Not possible, instead of proximity truncated phrases are advised[4]</td><td>(Mesh# "Osteoarthritis, Hip" OR AB,TI(Coxarthros* OR ((|) N/3 ())))</td></tr>
</table>

Journal of the Medical Library Association

Supplemental content to
J Med Libr Assoc. Oct;106(4):dx.doi.org/10.5195/jmla.2018.283
www.jmla.mlanet.org
© Bramer, de Jonge, Rethlefsen, Mast, Kleijnen 2018

Table 1 Using database-appropriate syntax, with parentheses, Boolean operators, and field codes (continued)

	Ovid	EBSCOhost	Embase.com	PubMed sensitive	PubMed specific	ProQuest
Paste or type the words in the proximity syntax	(exp "Osteoarthritis, Hip"/ OR (Coxarthros* OR ((Hip) ADJ3 (Osteoarthrit*))).ab,ti.)	(MH "Osteoarthritis, Hip+" OR AB (Coxarthros* OR ((Hip) N3 (Osteoarthrit*))))	('hip osteoarthritis'/exp OR (Coxarthros* OR 'malum coxae sinilis' OR ((hip OR cox) NEAR/3 (arthrit* OR arthros* OR osteoarthr*))):ab,ti)	("Osteoarthritis, Hip"[mh] OR (Coxarthros*[tiab] OR ((hip*[tiab]) AND (Osteoarthrit*[tiab]))))	("Osteoarthritis, Hip"[mh] OR (Coxarthros*[tiab] OR Hip Osteoarthrit*[tiab] OR Osteoarthritis Of Hip*[tiab]))	(Mesh# "Osteoarthritis, Hip" OR AB,TI(Coxarthros* OR ((hip) N/3 (osteoarthrit*))))
Follow the same steps for the other elements	(exp "Exercise Therapy"/ OR (((Exercise) ADJ3 (Therap*))).ab,ti.)	(MH "Exercise Therapy+" OR AB (((Exercise) N3 (Therap*))))	('kinesiotherapy'/exp OR (kinesiotherap* OR kinesitherap* OR ((exercise) NEAR/3 (technique* OR treat* OR therap*))):ab,ti)	("Exercise Therapy" [mh] OR (Exercise Therap*[tiab]))	("Exercise Therapy"[mh] OR (Exercise Therap*[tiab]))	(Mesh# "Exercise Therapy" OR AB,TI (((Exercise) N/3 (Therap*))))
Combine all elements into one search strategy	(exp "Osteoarthritis, Hip"/ OR (Coxarthros* OR ((Hip) ADJ3 (Osteoarthrit*))).ab,ti.) AND (exp "Exercise Therapy"/ OR (((Exercise) ADJ3 (Therap*))).ab,ti.)	(MH "Osteoarthritis, Hip+" OR AB (Coxarthros* OR ((Hip) N3 (Osteoarthrit*)))) AND (MH "Exercise Therapy+" OR AB (((Exercise) N3 (Therap*))))	('hip osteoarthritis'/exp OR (Coxarthros* OR 'malum coxae sinilis' OR ((hip OR cox) NEAR/3 (arthrit* OR arthros* OR osteoarthr*))):ab,ti) AND ('kinesiotherapy'/exp OR (kinesiotherap* OR kinesitherap* OR ((exercise) NEAR/3 (technique* OR treat* OR therap*))):ab,ti)	("Osteoarthritis, Hip"[mh] OR (Coxarthros*[tiab] OR ((hip*[tiab]) AND (Osteoarthrit*[tiab])))) AND ("Exercise Therapy"[mh] OR (Exercise Therap*[tiab]))	("Osteoarthritis, Hip"[mh] OR (Coxarthros*[tiab] OR Hip Osteoarthrit*[tiab] OR Osteoarthritis Of Hip*[tiab])) AND ("Exercise Therapy"[mh] OR (Exercise Therap*[tiab]))	(Mesh# "Osteoarthritis, Hip" OR AB,TI(Coxarthros* OR ((hip) N/3 (osteoarthrit*)))) AND (Mesh# "Exercise Therapy" OR AB,TI (((Exercise) N/3 (Therap*))))

The current location of the cursor is shown by |.

1. True proximity is not possible; PubMed does not allow proximity search. A very sensitive way is to search for words combined with "AND," because searching for exact phrases will not retrieve all relevant hits.

2. For our method, we advise starting with only AB as a field cod in EBSCOhost. EBSCOhost cannot combine multiple fields. Search terms need to be repeated, for example, TI ("hip osteoarthritis") OR AB ("hip osteoarthritis"). This is very time consuming. After optimization, copy the AB fields and also use TI().

3. In PubMed, this is not necessary as the field [tiab] has to be repeated after each synonym; however, for optimization, this will be useful.

4. In PubMed, placing an asterisk (*) after a phrase combines the word with the previous word(s) into a phrase. Do not use quotes and an asterisk, as when using quotes, truncation will be ignored.

JMLA Journal of the Medical Library Association

SC
Supplemental content to
J Med Libr Assoc. Oct;106(4):dx.doi.org/10.5195/jmla.2018.283
www.jmla.mlanet.org
© Bramer, de Jonge, Rethlefsen, Mast, Kleijnen 2018

11. Optimize the search

Table 2 Optimizing the search

	Ovid	EBSCOhost	Embase.com	PubMed sensitive	PubMed specific	ProQuest
Starting query	(exp "Osteoarthritis, Hip"/ OR (Coxarthros* OR ((Hip) ADJ3 (Osteoarthrit*))).ab,ti.) AND (exp “Exercise Therapy”/ OR (((Exercise) ADJ3 (Therap*))).ab,ti.)	(MH "Osteoarthritis, Hip+" OR AB (Coxarthros* OR ((Hip) N3 (Osteoarthrit*)))) AND (MH "Exercise Therapy+" OR AB (((Exercise) N3 (Therap*))))	('hip osteoarthritis'/exp OR (Coxarthros* OR 'malum coxae sinilis' OR ((hip OR cox) NEAR/3 (arthrit* OR arthros* OR osteoarthr*))):ab,ti) AND ('kinesiotherapy'/exp OR (kinesiotherap* OR kinesitherap* OR ((exercise) NEAR/3 (technique* OR treat* OR therap*))):ab,ti)	("Osteoarthritis, Hip"[mh] OR (Coxarthros*[tiab] OR ((hip*[tiab]) AND (Osteoarthrit*[tiab])))) AND ("Exercise Therapy"[mh] OR (Exercise Therap*[tiab]))	("Osteoarthritis, Hip"[mh] OR (Coxarthros*[tiab] OR Hip Osteoarthrit*[tiab] OR Osteoarthritis Of Hip*[tiab])) AND ("Exercise Therapy"[mh] OR (Exercise Therap*[tiab]))	(Mesh# "Osteoarthritis, Hip" OR AB,TI(Coxarthros* OR ((hip) N/3 (osteoarthrit*)))) AND (Mesh# "Exercise Therapy" OR AB,TI (((Exercise) N/3 (Therap*))))
Optimization first element: thesaurus terms NOT title abstract terms	(exp *"Osteoarthritis, Hip"/ NOT (Coxarthros* OR ((Hip) ADJ3 (Osteoarthrit*))).ab,ti.) AND (exp "Exercise Therapy"/ OR (((Exercise) ADJ3 (Therap*))).ab,ti.)	(MJ "Osteoarthritis, Hip+" NOT AB (Coxarthros* OR ((Hip) N3 (Osteoarthrit*)))) AND (MH "Exercise Therapy+" OR AB (((Exercise) N3 (Therap*))))	('hip osteoarthritis'/exp/mj NOT (Coxarthros* OR 'malum coxae sinilis' OR ((hip OR cox) NEAR/3 (arthrit* OR arthros* OR osteoarthr*))):ab,ti) AND ('kinesiotherapy''/exp OR (kinesiotherap* OR kinesitherap* OR ((exercise) NEAR/3 (technique* OR treat* OR therap*))):ab,ti)	("Osteoarthritis, Hip"[mj] NOT (Coxarthros*[tiab] OR ((hip*[tiab]) AND (Osteoarthrit*[tiab])))) AND ("Exercise Therapy"[mh] OR (Exercise Therap*[tiab]))	("Osteoarthritis, Hip"[mj] NOT (Coxarthros*[tiab] OR Hip Osteoarthrit*[tiab] OR Osteoarthritis Of Hip*[tiab])) AND ("Exercise Therapy"[mh] OR (Exercise Therap*[tiab]))	(MJMesh# "Osteoarthritis, Hip" NOT AB,TI(Coxarthros* OR ((hip) N/3 (osteoarthrit*)))) AND (Mesh# "Exercise Therapy" OR AB,TI (((Exercise) N/3 (Therap*))))

JMLA Journal of the Medical Library Association

SC
Supplemental content to
J Med Libr Assoc. Oct;106(4):dx.doi.org/10.5195/jmla.2018.283
www.jmla.mlanet.org
© Bramer, de Jonge, Rethlefsen, Mast, Kleijnen 2018

Table 2 Optimizing the search (continued)

	Ovid	EBSCOhost	Embase.com	PubMed sensitive	PubMed specific	ProQuest
Extra words added	(exp "Osteoarthritis, Hip"/ OR (Coxarthros* OR ((Hip OR cox) ADJ3 (Osteoarthrit* OR arthrit* OR arthros* OR oa))).ab,ti.) AND (exp "Exercise Therapy"/ OR (((Exercise) ADJ3 (Therap*))).ab,ti.)	(MH "Osteoarthritis, Hip+" OR AB (Coxarthros* OR ((Hip OR cox) N3 (Osteoarthrit* OR arthrit* OR arthros* OR oa)))) AND (MH "Exercise Therapy+" OR AB (((Exercise) N3 (Therap*))))	('hip osteoarthritis'/exp OR (Coxarthros* OR 'malum coxae sinilis' OR ((hip OR cox) NEAR/3 (arthrit* OR arthros* OR osteoarthr* OR oa))):ab,ti) AND ('kinesiotherapy'/exp OR (kinesiotherap* OR kinesitherap* OR ((exercise) NEAR/3 (technique* OR treat* OR therap*))):ab,ti)	("Osteoarthritis, Hip"[mh] OR (Coxarthros*[tiab] OR ((hip*[tiab] OR cox[tiab]) AND (Osteoarthrit*[tiab] OR arthrit*[tiab] OR arthros*[tiab] OR oa[tiab])))) AND ("Exercise Therapy"[mh] OR (Exercise Therap*[tiab]))	("Osteoarthritis, Hip"[mh] OR (Coxarthros*[tiab] OR Hip Osteoarthrit*[tiab] OR cox Osteoarthrit*[tiab] OR Hip Osteo arthrit*[tiab] OR "Hip oa"[tiab] OR Osteoarthritis Of Hip*[tiab] OR Osteoarthritis Of the Hip*[tiab] OR "oa of the Hip"[tiab])) AND ("Exercise Therapy"[mh] OR (Exercise Therap*[tiab]))	(Mesh# "Osteoarthritis, Hip" OR AB,TI(Coxarthros* OR ((hip OR cox) N/3 (osteoarthrit* OR arthrit* OR arthros* OR oa)))) AND (Mesh# "Exercise Therapy" OR AB,TI (((Exercise) N/3 (Therap*))))
Optimization second element: title terms NOT thesaurus terms	(exp "Osteoarthritis, Hip"/ OR (Coxarthros* OR ((Hip OR cox) ADJ3 (Osteoarthrit* OR arthrit* OR arthros* OR oa))).ab,ti.) AND ((((Exercise) ADJ3 (Therap*))).ti.) NOT (exp "Exercise Therapy"/)	(MH "Osteoarthritis, Hip+" OR AB (Coxarthros* OR ((Hip OR cox) N3 (Osteoarthrit* OR arthrit* OR arthros* OR oa)))) AND (TI(((Exercise) N3 (Therap*)))) NOT (MH "Exercise Therapy+")	('hip osteoarthritis'/exp OR (Coxarthros* OR 'malum coxae sinilis' OR ((hip OR cox) NEAR/3 (arthrit* OR arthros* OR osteoarthr* OR oa))):ab,ti) AND ((kinesiotherap* OR kinesitherap* OR ((exercise) NEAR/3 (technique* OR treat* OR therap*))):ti) NOT ('kinesiotherapy'/exp)	("Osteoarthritis, Hip"[mh] OR (Coxarthros*[tiab] OR ((hip*[tiab] OR cox[tiab]) AND (Osteoarthrit*[tiab] OR arthrit*[tiab] OR arthros*[tiab] OR oa[tiab])))) AND ((Exercise Therap*[ti])) NOT ("Exercise Therapy"[mh)	("Osteoarthritis, Hip"[mh] OR (Coxarthros*[tiab] OR Hip Osteoarthrit*[tiab] OR cox Osteoarthrit*[tiab] OR Hip Osteo arthrit*[tiab] OR "Hip oa"[tiab] OR Osteoarthritis Of Hip*[tiab] OR Osteoarthritis Of the Hip*[tiab] OR "oa of the Hip"[tiab])) AND ((Exercise Therap*[ti])) NOT ("Exercise Therapy"[mh)	(Mesh# "Osteoarthritis, Hip" OR AB,TI(Coxarthros* OR ((hip OR cox) N/3 (osteoarthrit* OR arthrit* OR arthros* OR oa)))) AND (TI (((Exercise) N/3 (Therap*)))) NOT (Mesh# "Exercise Therapy")

JMLA Journal of the Medical Library Association

Supplemental content to
J Med Libr Assoc. Oct;106(4):dx.doi.org/10.5195/jmla.2018.283
www.jmla.mlanet.org
© Bramer, de Jonge, Rethlefsen, Mast, Kleijnen 2018

Table 2 Optimizing the search (continued)

	Ovid	EBSCOhost	Embase.com	PubMed sensitive	PubMed specific	ProQuest
Extra words added<br><br>These steps should be performed repeatedly on all elements in the search strategy<br><br>Note: Only after all optimization is finished AB in the EBSCOhost should be copied for all elements into both AB() OR TI()	(exp "Osteoarthritis, Hip"/ OR (Coxarthros* OR ((Hip OR cox) ADJ3 (Osteoarthrit* OR arthrit* OR arthros* OR oa))).ab,ti.) AND (exp "Exercise Therapy"/ OR exp "Musculoskeletal Manipulations"/ OR exp "Exercise"/ OR exp "Physical Therapy Modalities"/ OR (((Exercise) ADJ3 (Therap*))).ab,ti.)	(MH "Osteoarthritis, Hip+" OR AB (Coxarthros* OR ((Hip OR cox) N3 (Osteoarthrit* OR arthrit* OR arthros* OR oa))) TI (Coxarthros* OR ((Hip OR cox) N3 (Osteoarthrit* OR arthrit* OR arthros* OR oa)))) AND (MH "Exercise Therapy+" OR MH "Musculoskeletal Manipulations+" OR MH "Exercise+" OR MH "Physical Therapy Modalities+" OR AB (((Exercise) N3 (Therap*))) OR TI (((Exercise) N3 (Therap*))))	('hip osteoarthritis'/exp OR (Coxarthros* OR 'malum coxae sinilis' OR ((hip OR cox) NEAR/3 (arthrit* OR arthros* OR osteoarthr* OR oa))):ab,ti) AND ('kinesiotherapy'/exp OR 'physiotherapy'/exp OR 'manipulative medicine'/exp OR exercise/exp OR (kinesiotherap* OR kinesitherap* OR ((exercise) NEAR/3 (technique* OR treat* OR therap*))):ab,ti)	("Osteoarthritis, Hip"[mh] OR (Coxarthros*[tiab] OR ((hip*[tiab] OR cox[tiab]) AND (Osteoarthrit*[tiab] OR arthrit*[tiab] OR arthros*[tiab] OR oa[tiab])))) AND ("Exercise Therapy"[mh] OR "Musculoskeletal Manipulations"[mh] OR "Exercise"[mh] OR "Physical Therapy Modalities"[mh] OR (Exercise Therap*[tiab]))	("Osteoarthritis, Hip" [mh] OR (Coxarthros*[tiab] OR Hip Osteoarthrit*[tiab] OR cox Osteoarthrit*[tiab] OR Hip Osteo arthrit*[tiab] OR "Hip oa" [tiab] OR Osteoarthritis Of Hip*[tiab] OR Osteoarthritis Of the Hip*[tiab] OR "oa of the Hip"[tiab])) AND ("Exercise Therapy"[mh] OR "Musculoskeletal Manipulations" [mh] OR "Exercise"[mh] OR "Physical Therapy Modalities"[mh] OR (Exercise Therap*[tiab]))	(Mesh# "Osteoarthritis, Hip" OR AB,TI(Coxarthros* OR ((hip OR cox) N/3 (osteoarthrit* OR arthrit* OR arthros* OR oa)))) AND (Mesh# "Exercise Therapy" OR Mesh# "Musculoskeletal Manipulations" OR Mesh# "Exercise" OR Mesh# "Physical Therapy Modalities" OR AB,TI (((Exercise) N/3 (Therap*))))

Supplemental content to
J Med Libr Assoc. Oct;106(4):dx.doi.org/10.5195/jmla.2018.283
www.jmla.mlanet.org
© Bramer, de Jonge, Rethlefsen, Mast, Kleijnen 2018

12. Evaluate the initial results

Table 3 Methods to increase sensitivity or specificity for a search strategy

Increase sensitivity	Increase specificity
Add extra terms	Delete less relevant terms
Extra words/synonyms can be found in the retrieved references as described in this article, for instance, by searching for articles that have the thesaurus terms, but lack the words in title or abstract.	When in doubt whether a term adds value, look at the extra retrieved references: Do they seem relevant? If not, delete these synonym(s).
Generalize specific elements	Specify more general elements
Potential relevant articles may be indexed with broader thesaurus terms, because in these articles, an overview of related topics has been described.	Choose a more specific thesaurus term. If the narrower terms from broad thesaurus terms cause noise, solve this by using the no explode function.
Drop less important elements	Add an extra element
Check what happens if each element is deleted from the search strategy. If the number of hits does not increase to above what can be handled, that element is not necessary and can be deleted. If the number of hits is too high, scan the extra, retrieved relevant references for potentially new, relevant synonyms.	Extra elements will reduce the number of hits, but will increase the chance of missed relevant references. Always check the references that would be missed by introducing this element for extra, relevant terms. This can be very time consuming.
Use floating subheadings	Combine subheadings with thesaurus terms
Searching with subheadings as a separate element (e.g., "neoplasms" [mh] AND "prevention and control" [sh]) will retrieve all references where the subheading is used in combination with other thesaurus terms.	Floating subheadings can retrieve many irrelevant results. This noise can be reduced by combining thesaurus terms with subheadings (such as "neoplasms/prevention and control"[mh]). If using such combinations, also combine the free-text words of these elements in phrases or proximity.
Broaden proximity (or replace with AND)	Narrower proximity or phrases (instead of AND)
It can be determined with great precision whether to use 3, 4, or 5 words in proximity. Changes are often minimal. If starting with 3, try broadening to 6, then to 10, 15, etc. Check for each step the extra, retrieved references on possible relevance. Decide which proximity gives the best results. Using "AND" instead of proximity, often retrieves much noise but can identify relevant references.	A combination of two or more free-text words with AND is very sensitive and can lead to much noise. This noise can be reduced by combining words of two elements into phrases or proximity searches.
Add (phrase) truncation or shorten word stems	Remove truncation or lengthen word stems
Always add truncation at the end of a word and use word stems as short as possible unless this leads to irrelevant results.	Sometimes truncated words or phrases result in noise. In that case, lengthen the word stem/phrase or do not truncate.
Remove filters	Add filters (language or date)
Filters should only be applied in the end of the search strategy, if the number of hits retrieved is too high or the noise is too much. The safest method is often to categorize the retrieved references by hand.	Restricting to language(s) may create bias in the retrieved references. So the best results are retrieved not limiting on language. If you want to restrict, only restrict to English, not to languages spoken by you or your team members.<br>Limit on date can be done for two reasons: (1) a thorough systematic review has been carried out and needs to be updated, or (2) one of the elements in the question is a new concept and did not exist before a certain date.

Supplemental content to
J Med Libr Assoc. Oct;106(4):dx.doi.org/10.5195/jmla.2018.283
www.jmla.mlanet.org
© Bramer, de Jonge, Rethlefsen, Mast, Kleijnen 2018

13. Check for errors

14. Translate to other databases

Systematic reviews in Erasmus MC search Embase.com, MEDLINE via Ovid, Web of Science, Cochrane CENTRAL, and Google Scholar. The results of the translation between databases are shown below.

Embase.com

('hip osteoarthritis'/exp OR (Coxarthros* OR 'malum coxae sinilis' OR ((hip OR cox) NEAR/3 (arthrit* OR arthros* OR osteoarthr* OR oa))):ab,ti) AND ('kinesiotherapy'/exp OR 'physiotherapy'/exp OR 'manipulative medicine'/exp OR exercise/exp OR (kinesiotherap* OR kinesitherap* OR ((exercise) NEAR/3 (technique* OR treat* OR therap*))):ab,ti)

MEDLINE Ovid

The macro translated the syntax of Embase into this Ovid syntax.

(hip osteoarthritis/ OR (Coxarthros* OR malum coxae sinilis OR ((hip OR cox) ADJ3 (arthrit* OR arthros* OR osteoarthr* OR oa))).ab,ti.) AND (kinesiotherapy/ OR physiotherapy/ OR manipulative medicine/ OR exercise/ OR (kinesiotherap* OR kinesitherap* OR ((exercise) ADJ3 (technique* OR treat* OR therap*))).ab,ti.)

Manually the Emtree terms are replaced by MeSH terms. If narrower terms have to be taken into account (such as in the MeSH term "Physical Therapy Modalities"), exp is placed before the MeSH term. This is different than in Emtree, where "Kinesiotherapy" is not a narrower term of physiotherapy; the MeSH term "Exercise Therapy" is found below "Physical Therapy Modalities"; therefore, it does not need to be added. When searched in MeSH, the Emtree term "Manipulative Medicine" is mapped to "Osteopathic Medicine," which is not the most relevant term. "Musculoskeletal Manipulations" is a better translation.

(Osteoarthritis, Hip/ OR (Coxarthros* OR malum coxae sinilis OR ((hip OR cox) ADJ3 (arthrit* OR arthros* OR osteoarthr* OR oa))).ab,ti.) AND (exp Physical Therapy Modalities/ OR exp Musculoskeletal Manipulations/ OR exp Exercise/ OR (kinesiotherap* OR kinesitherap* OR ((exercise) ADJ3 (technique* OR treat* OR therap*))).ab,ti.)

Cochrane CENTRAL

For a Cochrane search, the syntax of Embase.com is adapted by removing the Emtree terms from the search. Cochrane only contains MeSH terms, but only copies those from MEDLINE. Therefore, searching the Cochrane Library with MeSH terms, when MEDLINE has already been searched, will not retrieve new articles. The syntax of the Cochrane library for title or abstract terms is similar to that of Embase.com.

((Coxarthros* OR 'malum coxae sinilis' OR ((hip OR cox) NEAR/3 (arthrit* OR arthros* OR osteoarthr* OR oa))):ab,ti) AND ((kinesiotherap* OR kinesitherap* OR ((exercise) NEAR/3 (technique* OR treat* OR therap*))):ab,ti)

Web of Science

Using the macros, the syntax for Cochrane CETRAL is translated in a syntax for Web of Science.

TS=(((Coxarthros* OR "malum coxae sinilis" OR ((hip OR cox) NEAR/2 (arthrit* OR arthros* OR osteoarthr* OR oa)))) AND ((kinesiotherap* OR kinesitherap* OR ((exercise) NEAR/2 (technique* OR treat* OR therap*)))))

Supplemental content to
J Med Libr Assoc. Oct;106(4):dx.doi.org/10.5195/jmla.2018.283
www.jmla.mlanet.org
© Bramer, de Jonge, Rethlefsen, Mast, Kleijnen 2018

Google Scholar

The macros change the syntax of Cochrane CENTRAL to that of Google Scholar. Because of limitations in the length of the search to 256 characters each "OR" is replaced by a vertical line.

"Coxarthros|"malum coxae sinilis"|"hip|cox arthrit|arthros|osteoarthr|oa "kinesiotherap|kinesitherap|"exercise technique|treat|therap

The Google Scholar syntax has to be edited to make it work. Instead of proximity, we use the option to place an OR (|) in a quoted phrase. The macro placed some quotes in the correct location; however, some quotes are misplaced and have to be removed. Since Google Scholar does not allow truncation, each truncated word has to be completed.

Coxarthrosis|"malum coxae sinilis"|"hip|cox arthritis|arthrosis|osteoarthritis|oa" kinesiotherapy|kinesitherapy|"exercise technique|treatment|therapy"

Optional other databases for which macros are available are CINAHL (EBSCOhost) and Scopus.

CINAHL (EBSCOhost)

The syntax of MEDLINE Ovid is translated into a CINAHL EBSCOhost syntax using a macro.

(Osteoarthritis, Hip+ OR (Coxarthros* OR malum coxae sinilis OR ((hip OR cox) N2 (arthrit* OR arthros* OR osteoarthr* OR oa)))) AND (MH Physical Therapy Modalities+ OR MH Musculoskeletal Manipulations+ OR MH Exercise+ OR (kinesiotherap* OR kinesitherap* OR ((exercise) N2 (technique* OR treat* OR therap*))))

Some editing is necessary. MeSH terms have to be replaced by CINAHL headings, though there are many overlaps, because the CINAHL headings are largely based on the MeSH terms. Each thesaurus term should be preceded by the MH field code. The search terms for title abstract have to be copied and preceded once by TI and once by AB. Sadly, EBSCOhost is unable to search for the combined AB,TI fields.

(MH Osteoarthritis, Hip OR TI (Coxarthros* OR malum coxae sinilis OR ((hip OR cox) N2 (arthrit* OR arthros* OR osteoarthr* OR oa))) OR AB (Coxarthros* OR malum coxae sinilis OR ((hip OR cox) N2 (arthrit* OR arthros* OR osteoarthr* OR oa)))) AND (MH Physical Therapy Modalities+ OR MH Musculoskeletal Manipulations+ OR MH Exercise+ OR TI (kinesiotherap* OR kinesitherap* OR ((exercise) N2 (technique* OR treat* OR therap*))) OR AB (kinesiotherap* OR kinesitherap* OR ((exercise) N2 (technique* OR treat* OR therap*))))

Scopus

Using the macros, the syntax for Web of Science is translated in a syntax for Scopus.

TITLE-ABS-KEY(((Coxarthros* OR "malum coxae sinilis" OR ((hip OR cox) W/2 (arthrit* OR arthros* OR osteoarthr* OR oa)))) AND ((kinesiotherap* OR kinesitherap* OR ((exercise) W/2 (technique* OR treat* OR therap*)))))

15. Test and reiterate
